# 

**Published:** 1904-01-15

**Authors:** 


### CONTENTS.

The Midwinter National Meetings, - - - - - - 1

Professional Standards, - - *By N. S. Hoff, D.D.S.* 8

"We Made Better Dentists Then Than Now,"
*By D. R. Stubblefield, M.D., D.D.S.* 20

Toothsome Topics, - - *By R. B. Tuller, D.D.S.* 33

The International Congress, - *By Jas. Truman, D.D.S.* 39

Dentinal Anesthesia. - - - *By Dr. Thiesing.* 43

Editorial, - - - - - - - - - 44

Announcements, - - - - - - - - 45

Obituary, - - - - - - - - - 46

Indents, - - - - - - - - - - 50

